# Pectopexy vs sacrocolpopexy: an analysis of 50 cases in a North American hospital

**DOI:** 10.1016/j.xagr.2023.100254

**Published:** 2023-07-17

**Authors:** John Heusinkveld, Maithili Khandekar, Veronica Winget, Alyssa Tigner, Ilana Addis

**Affiliations:** 1 Department of Obstetrics and Gynecology, University of Arizona, Tucson, AZ (Drs Heusinkveld, Winget, Tigner, and Addis); 2 University of Arizona College of Medicine, Tucson, AZ (Ms Khandekar).

**Keywords:** laparoscopic surgery, minimally invasive surgery, pelvic organ prolapse, vaginal vault prolapse

## Abstract

**BACKGROUND:**

Laparoscopic pectopexy is an alternative to sacrocolpopexy that was first reported in 2010. This procedure has been performed at our hospital since 2019 in patients with contraindications to sacrocolpopexy.

**OBJECTIVE:**

This study aimed to compare the outcomes of 50 cases of pectopexy with historical outcomes data for sacrocolpopexy.

**STUDY DESIGN:**

This was a retrospective review of 50 laparoscopic pectopexies performed from July 2020 to July 2022 at an academic tertiary referral center; this was the second reported use of this technique in North America. The outcomes from laparoscopic pectopexy were compared with laparoscopic sacrocolpopexy performed at the same institution by the same surgeons (n=207). The primary outcomes were complication rate, rate of recurrent prolapse (stage II or greater), and reoperation.

**RESULTS:**

Overall complication rates were 6.0% for pectopexy and 16.5% for sacrocolpopexy (relative risk, 0.79; *P*=.65). Recurrent prolapse was seen among 2.0% of patients who underwent pectopexy and 6.3% of patients who underwent sacrocolpopexy at most recent follow-up (relative risk, 1.27; *P*=.66). The rates of reoperation were 2.0% for pectopexy and 3.9% for sacrocolpopexy (relative risk, 1.04; *P*=.96). The average operative times were 138 minutes for pectopexy and 158 minutes for sacrocolpopexy. The average lengths of follow-up were 88.1 days for pectopexy and 325.5 for sacrocolpopexy.

**CONCLUSION:**

Although pectopexy was typically employed in patients with extensive pelvic adhesions or other conditions that placed them at higher risk of complications, both the success rate and the adverse event rate were similar to those in the historical cohort who underwent sacrocolpopexy. Although sacrocolpopexy remains the gold standard operation for apical prolapse, our data suggest that pectopexy can be employed to offer similar outcomes in many patients with contraindications to sacral fixation. These data give us increasing confidence that we can counsel our patients that this operation is likely to produce an outcome similar to a sacrocolpopexy.

**AJOG Global Reports at a Glance**

**Why was this study conducted?**

This study aimed to compare the outcomes between laparoscopic pectopexy and laparoscopic sacrocolpopexy.

**Key findings**

The success rate and complication rate were similar between laparoscopic sacrocolpopexy and laparoscopic pectopexy, although pectopexy was often used in more complex patients.

**What does this add to what is known?**

This study supports pectopexy as an alternative for vaginal vault support in patients who have contraindications to sacrocolpopexy.

## Introduction

Sacrocolpopexy is widely regarded as the gold standard operation for apical prolapse, based on several observational studies, prospective trials, and a meta-analysis by the Cochrane organization, which show superior results compared with vaginal interventions with an acceptable rate of complications, including those related to the use of artificial mesh.^1^ However, some aspects of the operation create higher risks in patients with certain conditions. The operation is notoriously difficult in patients with extensive intra-abdominal adhesions, especially when they involve the colon and the deep pelvis, and conversion from a minimally invasive approach to laparotomy is often necessary for such situations. In patients with extensive diverticulosis, a fairly common condition in the age group who are at highest risk of prolapse, placement of mesh close to the colon has been reported as a risk factor for mesh erosion into the colon.^2^

**Figure 1:**
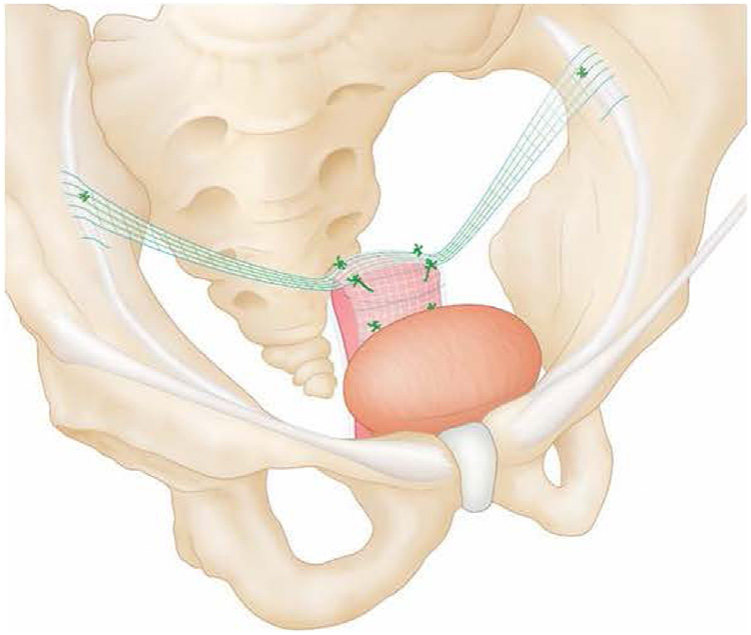
Pectopexy illustration

Pectopexy is an alternative to sacrocolpopexy, which was first described by Noé in 2010.^3^ Similar to sacrocolpopexy, pectopexy uses a macroporous, monofilament mesh, which is attached to the anterior and/or posterior wall of the vagina with arms that attach to the right and left pectineal ligaments instead of the presacral ligament (Figure 1). Pectopexy has theoretical advantages for patients with several conditions, which may place them at higher risk of complications with sacrocolpopexy. The successful use of the pectopexy technique in North America was first reported in 2021 in a small case series.^4^ In a randomized trial in Germany comparing pectopexy with sacrocolpopexy, equivalent outcomes and complications were observed with fewer bowel complications in the pectopexy cohort.^5^

Heusinkveld. Pectopexy vs sacrocolpopexy. Am J Obstet Gynecol Glob Rep 2023.

Our group has been performing pectopexy in select patients since late 2019. We analyzed the results of 50 cases performed from July 2021 to July 2022 and compared the results with historical data from 206 sacrocolpopexies performed from 2014 to 2020 to determine how the results of the new operation compare with the gold standard operation.

## Materials and Methods

Under an institutional review board–approved protocol, 50 cases of pectopexy performed between July 2020 and July 2021 were reviewed. Sacrocolpopexy remains a first-line treatment of recurrent apical prolapse at our institution, and all patients who underwent pectopexy did so because of a perceived contraindication to sacrocolpopexy. All patients consented to both procedures, and the decision to perform pectopexy instead of sacrocolpopexy is based on preoperative risk factors and intraoperative findings, including pelvic adhesive disease, colonic pathology, bleeding risk, and frailty.

We perform sacrocolpopexy via a 5-port technique with an umbilical camera port and 2 lateral ports on each side so that both surgeons can operate with both hands. We use an ultralightweight type 1 polypropylene Y-mesh, as is the current standard of care, sutured to the vagina with polytetrafluoroethylene and to the presacral ligament with polyester.

Our technique for pectopexy is shown in the Video. We use the same port configuration as for sacrocolpopexy. A vaginal manipulator is placed, and a plane is developed between the bladder and the vagina using sharp dissection to permit attachment of a 4-cm anterior mesh strap. In patients without a uterus, the peritoneum is removed from a similar length of the posterior vaginal wall. The peritoneal dissection is extended laterally to expose the right and left pectineal ligaments, which are found beneath the obliterated umbilical arteries.

Subsequently, a sacrocolpopexy Y-mesh is modified by dividing the sacral strap down the middle and trimming the anterior and posterior straps to fit the denuded areas of the anterior and posterior vaginal wall. In patients with an intact uterus, the posterior strap is removed. The anterior and posterior strap, if present, are sewn to the vaginal walls with 3 polytetrafluoroethylene sutures on each side. The right half of the divided sacral strap is sewn to the right pectineal ligament with polyester, and the same procedure is performed on the left side. Finally, the peritoneum is closed over the mesh to prevent contact with the bowel.

## Results

Patient characteristics are summarized in Table 1 and were similar between the 2 groups. The overall complication rates were 6.0% for pectopexy and 16.5% for sacrocolpopexy (relative risk [RR], 0.79; *P*=.65). The rates of recurrent prolapse were 2.0% for pectopexy and 6.3% for sacrocolpopexy (RR, 1.27; *P*=.66), and the rates of reoperation were 2.0% for pectopexy and 3.9% for sacrocolpopexy (RR, 1.04; *P*=.96). The average operative times were 138 minutes for pectopexy and 158 minutes for sacrocolpopexy. The average lengths of follow-up were 325.5 days (range, 10–2198) for sacrocolpopexy and 88.14 days (range, 0–369) for pectopexy.

**Table 1:** Patient characteristics in laparoscopic sacrocolpopexy and pectopexy

Demographic factor	Laparoscopic sacrocolpopexy, mean (SD; range)	Pectopexy, mean (SD; range)
Age	64.9 (10.3; 21–92)	69.7 (9.8; 43–87)
Body mass index	27.9 (4.5; 19.00–37.00)	28.4 (5.7; 18.68–45.00)
Vaginal deliveries	2.6 (1.5; 0–12)	2.4 (1.4; 0–5)
Prolapse stage (POP-Q)	2.60 (0.66; 1–4)	2.76 (0.50; 2–4)
Failed previous prolapse surgery	69 (33.5%)	8 (16.0%)

*POP-Q*, Pelvic Organ Prolapse Quantification; *SD*, standard deviation.

**Figure 2:**
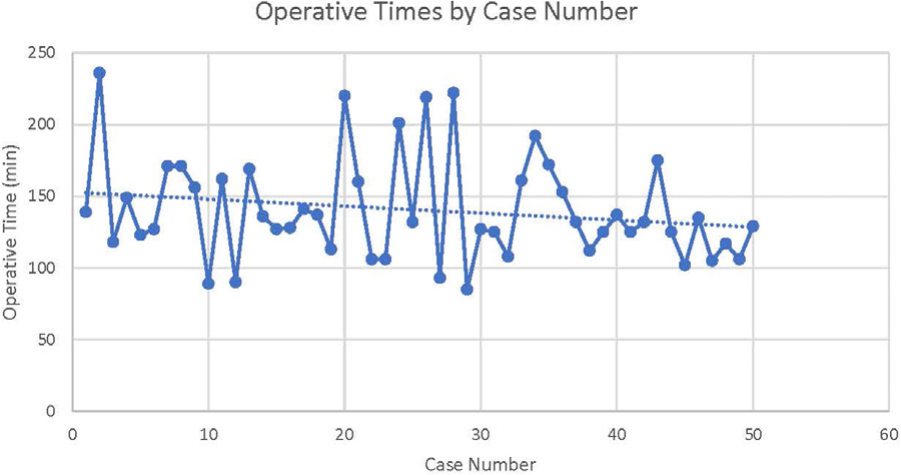
Operative times by case number

Operative times were relatively stable throughout the study period (Figure 2). The rates of complications and prolapse recurrence were similar between the two groups (Table 2).

Heusinkveld. Pectopexy vs sacrocolpopexy. Am J Obstet Gynecol Glob Rep 2023.

**Table 2:** Complications in pectopexy cohort vs sacrocolpopexy cohort

Complications	Laparoscopic sacrocolpopexy	Pectopexy
Hematoma	2 (1.0)	0 (0)
Mesh complications (erosion, detachment, removal)	5 (2.4)	0 (0)
Recurrent prolapse (POP-Q of >2)	13 (6.3)	1 (2.0)
Prolapse reoperation	8 (3.9)	1 (2.0)
Urinary retention requiring intervention	2 (1.0)	1 (2.0)
Overall complication rate	34 (16.5)	3 (6.0)
Average follow-up (d), median (IQR)	325.50 (10–2198)	88.14 (0–369)

*IQR*, interquartile range; *POP-Q*, Pelvic Organ Prolapse Quantification.

## Discussion

### Principal findings

Our data showed that the short-term outcomes of laparoscopic pectopexy are similar to those that we have historically obtained with sacrocolpopexy. Although we tend to use this operation in complex patients who have relative contraindications to sacrocolpopexy, the rate of complications in patients who underwent pectopexy was similar to that of patients who underwent sacrocolpopexy.

### Results

Although the rates of recurrent prolapse and reoperation were similar, the results must be interpreted with caution because of the limited duration of follow-up on patients who underwent pectopexy. It is possible that prolapse recurrences and reoperations among patients who undergo pectopexy will increase with longer follow-up, although this has not occurred in previous randomized controlled trials (RCTs).^5^

### Clinical implications

Sacrocolpopexy requires dissection of the presacral space, increasing the risk of intraoperative hemorrhage, especially among elderly patients and those on anticoagulation. The same patients are more susceptible to adverse effects of fluid shifts associated with such bleeding events and their treatment. In addition, sacrocolpopexy results in the placement of mesh directly adjacent to the colon, which can lead to higher risk in patients with colonic pathology. Finally, exposure of the deep pelvis requires an extended period in the steep Trendelenburg position, challenging both ventilation and circulation.

Because the entire pectopexy operation takes place in the anterior pelvis, dissection in the deep pelvis is avoided in patients with extensive adhesions, and we have not needed to convert any patients to an open approach as we became proficient in performing pectopexy. Similarly, the need for dissection of the presacral space, with its consequent bleeding risk, is avoided in patients requiring anticoagulation and those at high risk of adverse events related to blood loss. Moreover, the location of the operation exclusively in the anterior pelvis allows for a less steep Trendelenburg position, which may be beneficial for patients with respiratory or circulatory compromise. The placement of the mesh relatively distant from the colon may also reduce the chance of colonic mesh erosion or extensive adhesions, which could complicate future surgery for colonic pathology.

Together with previous RCT data and case series from other institutions, the results give us increased confidence that we can counsel our patients that pectopexy is likely to produce results similar to sacrocolpopexy with a similar risk of complications. Based on the fact that the techniques use the same materials and suturing methods and differ only in the choice of fixation point, we are optimistic that pectopexy will have a long-term success rate similar to sacrocolpopexy, although this will need to be confirmed with longer-term follow-up of this cohort of patients. We continue to regard sacrocolpopexy, which has been performed for more than 50 years and which our group has performed more than 500 times, as the gold standard to be used unless a contraindication exists; however, we regard pectopexy as a useful addition to our toolbox, which seems to offer similar outcomes to patients who are not ideal candidates for sacrocolpopexy.

### Research implications

More longitudinal data are needed on pectopexy, in the form of both observational research studies and prospective trials. Because of the different indications for sacrocolpopexy and pectopexy at this time, enrollment in an RCT would be challenging and limited to patients who are judged to be equally suitable candidates for either procedure, which, in our practice, is not a large set of patients. However, because most published data on sacrocolpopexy are observational, the publication of similar data on pectopexy should allow meta-analyses over time to answer some of the questions that would allay the difficulty of performing an RCT.

For this preliminary study, focused primarily on safety, we chose for comparison a group of patients who underwent sacrocolpopexy before pectopexy was routinely offered in our practice, because we believe that the fact that we tend to use pectopexy in more surgically complex patients may lead to a higher complication rate in the pectopexy cohort than in the sacrocolpopexy cohort. An intermediate-term follow-up study is currently in progress, which will include a 2- to 3-year follow-up on all patients who underwent prolapse surgery during the 1-year period over which these data were obtained. This study will provide a clearer picture of how the efficacy of pectopexy compares with other prolapse operations.

### Strengths and limitations

The strengths of our study include a relatively large cohort compared with other published studies on pectopexy, a large comparison group with longer follow-up, and the use of clinically meaningful endpoints of complications, prolapse recurrence, and reoperation.

The limitations of our study include the observational nature of the study, the limited length of follow-up on the patients who underwent pectopexy, and the fact that heterogeneity in the sacrocolpopexy data because of variations in the surgeons’ documentation styles prevents a detailed comparison of anatomic outcomes, although current evidence does not support a correlation between “superior” anatomic outcomes and patient satisfaction.^6^

### Conclusions

In a cohort of patients with relative contraindications to sacrocolpopexy, pectopexy seemed to produce similar results to those which we have traditionally obtained with sacrocolpopexy. Our data support the use of pectopexy among patients with relative contraindications to sacrocolpopexy.

## Appendix Supplementary materials

**Video** Procedure for pectopexy Heusinkveld. Pectopexy vs sacrocolpopexy. Am J Obstet Gynecol Glob Rep 2023. Image, video 1

## References

[1] Maher C, Feiner B, Baessler K, Christmann-Schmid C, Haya N, Brown J (2016). Surgery for women with apical vaginal prolapse. Cochrane Database Syst Rev.

[2] Lin X, Du P, Chen L, Gan Y, Zhang X (2018). A case of mesh erosion to the sigmoid after laparoscopic sacrocolpopexy and a literature review of mesh related complications. Female Pelvic Med Reconstr Surg.

[3] Banerjee C, Noé KG (2011). Laparoscopic pectopexy: a new technique of prolapse surgery for obese patients. Arch Gynecol Obstet.

[4] Winget VL, Gabra MG, Addis IB, Hatch KK, Heusinkveld JM (2021). Laparoscopic pectopexy for patients with intraabdominal adhesions, lumbar spinal procedures, and other contraindications to sacrocolpopexy: a case series. AJOG Glob Rep.

[5] Noé GK, Anapolski M (2014). A randomized trial of laparoscopic sacral colpopexy versus laparoscopic pectopexy for vaginal and uterine prolapse. J Minim Invasive Gynecol.

[6] Jelovsek JE, Gantz MG, Lukacz E (2021). Success and failure are dynamic, recurrent event states after surgical treatment for pelvic organ prolapse. Am J Obstet Gynecol.

